# Genome-wide association studies identified multiple genetic loci for body size at four growth stages in Chinese Holstein cattle

**DOI:** 10.1371/journal.pone.0175971

**Published:** 2017-04-20

**Authors:** Xu Zhang, Qin Chu, Gang Guo, Ganghui Dong, Xizhi Li, Qin Zhang, Shengli Zhang, Zhiwu Zhang, Yachun Wang

**Affiliations:** 1 Key Laboratory of Agricultural Animal Genetics and Breeding, National Engineering Laboratory for Animal Breeding, College of Animal Science and Technology, China Agricultural University, Beijing, P.R. China; 2 Department of Crop and Soil Sciences, Washington State University, Pullman, Washington, United States of America; 3 Institute of Animal Husbandry and Veterinary Medicine, Beijing Academy of Agriculture and Forestry Sciences, Beijing, P.R. China; 4 Beijing Sunlon Livestock Development Co. Ltd, Beijing, P.R. China; Xiamen University, CHINA

## Abstract

The growth and maturity of cattle body size affect not only feed efficiency, but also productivity and longevity. Dissecting the genetic architecture of body size is critical for cattle breeding to improve both efficiency and productivity. The volume and weight of body size are indicated by several measurements. Among them, Heart Girth (HG) and Hip Height (HH) are the most important traits. They are widely used as predictors of body weight (BW). Few association studies have been conducted for HG and HH in cattle focusing on single growth stage. In this study, we extended the Genome-wide association studies to a full spectrum of four growth stages (6-, 12-, 18-, and 24-months after birth) in Chinese Holstein heifers. The whole genomic single nucleotide polymorphisms (SNPs) were obtained from the Illumina BovineSNP50 v2 BeadChip genotyped on 3,325 individuals. Estimated breeding values (EBVs) were derived for both HG and HH at the four different ages and analyzed separately for GWAS by using the Fixed and random model Circuitous Probability Unification (FarmCPU) method. In total, 27 SNPs were identified to be significantly associated with HG and HH at different growth stages. We found 66 candidate genes located nearby the associated SNPs, including nine genes that were known as highly related to development and skeletal and muscular growth. In addition, biological function analysis was performed by Ingenuity Pathway Analysis and an interaction network related to development was obtained, which contained 16 genes out of the 66 candidates. The set of putative genes provided valuable resources and can help elucidate the genomic architecture and mechanisms underlying growth traits in dairy cattle.

## Introduction

Early development of heifer is important to the subsequent performance of a dairy herd because well-grown heifers probably calve and begin producing milk sooner, which do increase profit[1]. For dairy farms, heifer growth is routinely monitored for breeding selection[2] and management. Body weight (BW) has been reported as one of the measurements to determine the early growth in dairy farm[3]. The process of collecting routine BW measurements is labor intensive. Alternatively, measuring heifer body size was frequently selected by dairy farm managers to monitor growth and track the developmental progress of each animal throughout the rearing period[2].

Heart girth (HG) and hip height (HH) are the two major characteristics of body size in cattle. It is much easier to measure HG and HH than measuring BW. Both HG and HH are high heritable and have high phenotypic and genetic correlations with BW[4]. The prediction of BW based on HG and HH is regarded as highly reliable and accurate[5]. In addition, HG and HH are known having a relationship with important economic traits. For example, good depth of HG in cattle is a sign of good forage convertibility and good feet and leg conformation[6]. Heifers with higher HH will subsequently produce more milk[7]. It is widely accepted that HG and HH are quantitative traits, which are probably controlled by multiple genes[8]. Previous studies using marker association analysis[9–11], have reported some genes related to HG and HH in cattle. Recently, genome-wide association (GWAS) have revealed many important findings associated with production traits[12–14], growth traits[15,16], fertility traits[17,18] etc. in cattle. For HG and HH, many studies focus on only one growth stage, such as post-weaning height[19], yearling height[20], and stature in adulthood[13,21]. However, to date, no GWAS has been performed to evaluate the genetic architecture of HG and HH through the whole growing period in cattle.

In Chinese Holstein cattle, HG and HH from birth to first calving have become routine measurements during the past 20 years, which provides valuable resources to study the complete growing period. Thus, the purpose of our study was to provide new information of genetic markers related to HG and HH through the entire growth and development process by using the GWAS approach in dairy cattle.

## Materials and methods

Blood samples used in this study were collected along with the regular quarantine inspection in dairy farms, so no ethical approval was required.

### Experimental animals

In this study, 3 datasets were involved (Table 1). First, HG and HH phenotypic data were collected on 38,602 Chinese Holstein cows born between 2002 and 2011, and raised on 27 dairy farms in Beijing, China. Second, to obtain reliable breeding value estimates, the available pedigree information was traced back to the 1960s. All estimated breeding values (EBVs) of 73,806 cattle in the pedigree, including the 38,602 cows, were calculated. At last, a total of 3,325 individuals out of the 73,806 cattle in pedigree, consisting of 2,942 cows and 383 bulls, were genotyped.

**Table 1:** Structures of the three types of datasets used in this study.

Dataset	No. of bulls	No. of cows	Total	Interval of birth
Phenotypic data	0	38,602	38,602	2002–2011
Pedigree data	2,006	71,800	73,806	1965–2011
Genotypic data	383	2,942	3,325	1965–2011

### Phenotypic data

For each cow, HG and HH were measured simultaneously. Totally, 90,664 HG and 90,664 HH records from 38,602 cows (Table 1) were obtained, with an average of 3 records per cow. These measurements were collected roughly around 6, 12, 18 and 24 months, but not exactly across all the individuals, as shown in S1 Fig (RAW). Therefore, an adjustment was implemented firstly, using the Gompertz curve[22] modeled as follows:

$$HG_{age}=222.8\text{exp}\left(-0.8839\text{exp}\left(-0.002932age\right)\right)\;\left(r^{2}=0.973\right)$$ (1)

$$HH_{age}=144.1\text{exp}\left(-0.5856\text{exp}\left(-0.003522age\right)\right)\;\left(r^{2}=0.890\right)$$ (2)

Then, the adjusted coefficients were calculated to adjust the HG and HH to four age categories: 6, 12, 18, and 24 months, which corresponded to the measurements in 2–10, 8–16, 14–22, 20–28 months old, respectively. If two or more measurements from the same individual were contained in the same category, only the one closest to the adjusted month was kept. The basic statistics of the adjusted HG and HH were listed in Table 1. In this study, HG6, HH6, HG12, HH12, HG18, HH18, HG24, and HH24, were treated as eight traits for the subsequent analysis, as shown in S1 Fig (ADJUSTED).

### Estimated breeding values

Using the adjusted age-trait phenotypes, we calculated the EBVs of HG and HH for all 73,806 cattle in the pedigree (Table 1).

We used a multiple-trait animal model in the genetic evaluation, as follows:

$$y_{ijk}=\mu +Herd_{i}+a_{jk}+e_{ijk}$$ (3)

where *y*_*ijk*_ is the phenotype for the *j*^th^ cow from the *i*^th^ herd for the *k*^th^ trait (age-adjusted HG or HH); *μ* is the overall mean; *Herd*_*i*_ is the effect of the *i*^th^ herd; *a*_*jk*_ is the additive genetic effect of the *j*^th^ cow and the *k*^th^ trait; and *e*_*ijk*_ is the vector of residual effects.

The DMU software[23] was used to estimate the EBVs. The Average Information Restricted Maximum Likelihood (AI-REML)[24] was applied to the variance components estimation. Furthermore, the EBVs of each trait were tested for normality of distribution by the Shapiro-Wilk Normality Test[25].

### Genotypic data

Blood samples of 2,942 cows and semen samples of 383 bulls (Table 1) were used to extract the DNA by routine procedures. After that, the DNAs were quantified and genotyped using the BovineSNP50 v2 (Illumina, Inc., San Diego CA). The rate of missing genotypes was lower than 10% in all individuals. The SNPs were removed from dataset if it exhibited: 1) a call rate less than 90%; 2) minor allele frequency (MAF) less than 5%; 3) Fisher’s exact test *p*-value for Hardy-Weinberg Equilibrium (HWE) greater than or equal to 1 × 10^−6^; and 4) chromosome and position were unknown.

**Fig 1:**
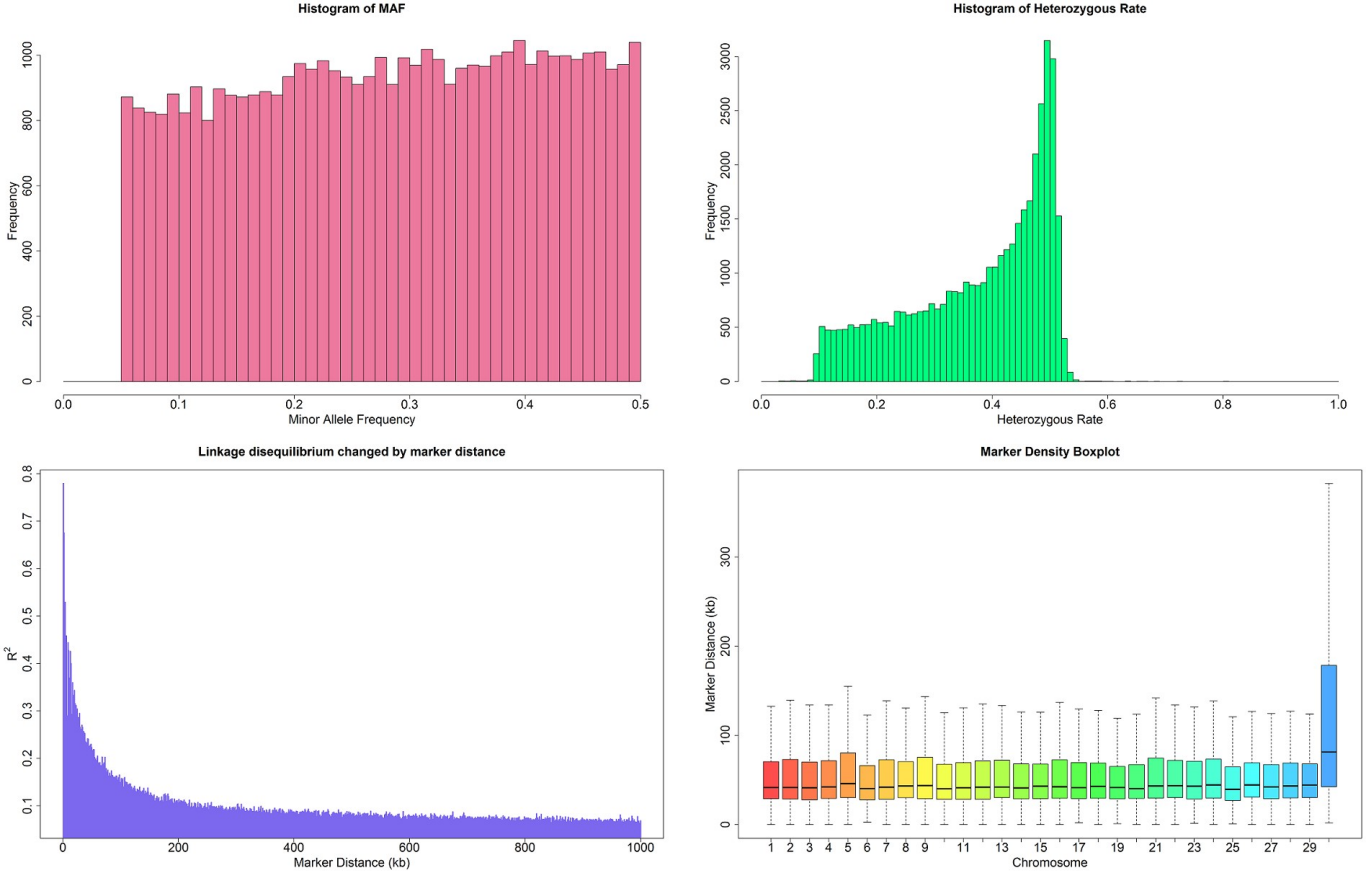
Characteristics of genotypes in Chinese Holstein cattle. **A) The distribution of the Minor Allele Frequency (MAF).** All the SNPs with MAF < 0.05 were removed from the analysis. For the remaining SNPs, frequency increases incrementally with increasing MAF. B) The heterozygous rate distribution. Most of the SNPs have a medium rate of heterozygous. The heterozygous rate of about 50% exhibited the highest frequency. C) Linkage disequilibrium (LD) change by marker distance. The average LD between two markers with a distance of less than 10 kb is more than 0.6. R-square decreased quickly with increasing marker distance. D) The average marker distance in the 30 bovine chromosomes. Chromosome 1–29 are autosomes; X is the sex chromosome. The average marker distance in all autosomes is about 50 kb. The marker distance in the sex chromosome is almost 100 kb.

Of the total 54,609 SNPs, 42,307 on the 30 bovine chromosomes satisfied these selection criteria. The number of SNPs varied among chromosomes, with Bos Taurus autosome 1(BTA1) having the largest number of SNPs (2,711) and BTA 27 and BTA 28 having the fewest number of SNPs (778). The distribution of the minor allele frequency (MAF), heterozygous rate, linkage disequilibrium (LD) changed by marker distance and the average marker distance were shown in Fig 1.

### Association analysis

The Fixed and random model Circuitous Probability Unification (FarmCPU) model[26] was used to perform the single-SNP analysis for the markers. Initially, a permutation test was applied to determine the significant threshold for the first iteration in FarmCPU. Subsequently, Bonferroni-corrected threshold probability of 0.05/N was implemented to verify the significance levels for the GWAS results, where N was the number of trait-SNP combinations tested. Quantile-quantile (Q-Q) plots and Manhattan plots were created by R 3.1.1 software[27].

### Identification and annotation of candidate genes

Gene identification information was downloaded from the UC Santa Cruz genome annotation database (http://hgdownload.soe.ucsc.edu/goldenPath/bosTau6/database/) for the Nov. 2009 (Bos_taurus_UMD_3.1/bosTau6). Ingenuity Pathway Analysis (IPA) was performed to identify the potential pathway, network and functional annotation of candidate genes.

## Results

### Genetic parameters of the traits

Using the multiple-trait model, the variance components and the genetic parameters for each HG and HH trait were estimated (Tables 2 and 3). Obviously, both HH and HG are highly heritable, with similar heritability estimates. The heritabilities for HG varied from 0.33±0.019 to 0.40±0.018, while those for HH ranged from 0.35±0.020 to 0.40±0.036.

**Table 2:** Statistics for adjusted measurements of 38,602 individuals at 6, 12, 18, and 24 months after birth.

Trait	Age	N	Mean	S.D.	$\sigma_{a}^{2}$	$\sigma_{p}^{2}$
Heart girth (cm)	6	24, 951	133.61	7.520	14.87 (0.948)	45.13 (1.213)
	12	28, 667	165.04	8.192	17.05 (0.876)	42.85 (1.103)
	18	22, 651	181.69	8.610	16.09 (1.014)	45.46 (1.291)
	24	8, 341	203.58	8.312	16.42 (1.815)	46.93 (2.338)
Hip height (cm)	6	24, 951	105.94	4.305	4.81 (0.306)	13.79 (0.390)
	12	28, 667	122.15	4.201	4.69 (0.252)	12.25 (0.318)
	18	22, 651	131.89	4.286	4.86 (0.291)	12.93 (0.369)
	24	8, 341	138.02	4.301	5.18 (0.514)	12.95 (0.657)

Age: age of month

N: number of adjusted-records

S.D.: standard deviation

$\sigma_{a}^{2}$: the additive variance, with their standard errors (in parenthesis)

$\sigma_{p}^{2}$: the phenotypic variance, with their standard errors (in parenthesis)

**Table 3:** Heritabilities, genetic and phenotypic correlations for Heart Girth (HG) and Hip Height (HH) in Chinese Holstein.

**Trait**	**HH6**	**HG6**	**HH12**	**HG12**	**HH18**	**HG18**	**HH24**	**HG24**
HH6	0.35 (0.020)	0.47 (0.006)	0.50 (0.007)	0.30 (0.007)	0.47 (0.008)	0.28 (0.008)	0.43 (0.013)	0.26 (0.014)
HG6	0.64 (0.001)	0.33 (0.019)	0.24 (0.007)	0.43 (0.007)	0.25 (0.009)	0.42 (0.008)	0.24 (0.014)	0.35 (0.013)
HH12	0.56 (0.001)	0.36 (0.002)	0.38 (0.018)	0.46 (0.005)	0.71 (0.005)	0.35 (0.006)	0.50 (0.011)	0.29 (0.013)
HG12	0.34 (0.002)	0.43 (0.002)	0.65 (0.001)	0.40 (0.018)	0.41 (0.006)	0.75 (0.005)	0.34 (0.012)	0.50 (0.011)
HH18	0.54 (0.002)	0.36 (0.002)	0.90 (0.001)	0.57 (0.001)	0.38 (0.020)	0.44 (0.006)	0.57 (0.012)	0.33 (0.013)
HG18	0.30 (0.002)	0.39 (0.002)	0.58 (0.001)	0.81 (0.001)	0.57 (0.001)	0.35 (0.020)	0.34 (0.013)	0.51 (0.012)
HH24	0.62 (0.003)	0.45 (0.003)	0.67 (0.002)	0.43 (0.003)	0.63 (0.003)	0.43 (0.004)	0.40 (0.036)	0.44 (0.010)
HG24	0.40 (0.004)	0.35 (0.004)	0.35 (0.004)	0.53 (0.003)	0.36 (0.004)	0.56 (0.004)	0.60 (0.003)	0.35 (0.035)

The number neighboring the trait indicates the adjusted-age of measurement, e.g., HG6 = heart girth at 6 months.

Heritabilities (on diagonal), genetic correlations (below the diagonal) and phenotypic correlations (above the diagonal) were derived using a multiple-trait animal model.

Estimates in parenthesis were the standard errors.

It is obvious that the phenotypic correlations were commonly higher between the same traits at different ages compared with the correlations between different traits. The phenotypic correlations between HG traits ranged from 0.35±0.013 to 0.75±0.005, while those between HH traits varied from 0.43±0.013 to 0.71±0.05. By contrast, the phenotypic correlations between HG and HH traits were lower, ranging from 0.24±0.007 to 0.47±0.006.

The genetic correlations have a similar trend as the phenotypic correlations. The genetic correlations between HG traits ranged from 0.35±0.004 to 0.81±0.001, and those between HH traits ranged from 0.54±0.002 to 0.90±0.001. The genetic correlations between HG and HH traits ranged from 0.30±0.002 to 0.65±0.001. The phenotypic and genetic correlations between HG12 and HG18 were 0.75 and 0.81, respectively, which were the highest among HG between different age categories. Interestingly, for HH, the highest phenotypic and genetic correlations were also from the correlation between HH12 and HH18, with the estimates of 0.71 and 0.90, respectively.

### GWAS

**Fig 2:**
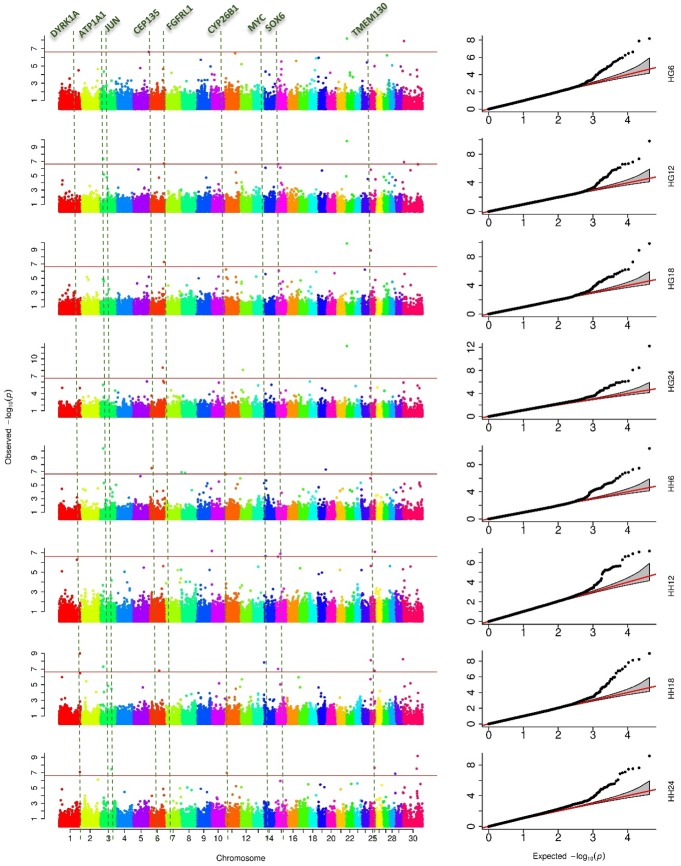
Results of the genome-wide association studies. The strengths of genome-wide association studies (GWAS) are illustrated by the Manhattan plots on the left panel. The deviations of the signals from null hypothesis are illustrated as the Quantile-Quantile (QQ) plots on the right panel. The negative logarithms of the observed (y axis) and the expected (x axis) P values are plotted for each SNP (dot). GWAS were performed on HG and HH at age of 6, 12, 18, and 24 months separately. Each analysis is labeled as trait (HG or HH) and month on the far right. The number neighboring each trait indicates the age of measurement (e.g., HG6 = Heart Girth at 6 months). The dependent variables were the estimated breeding values (EBVs) calculated from 3,325 individuals, which were genotyped with 42,307 SNPs. The horizontal red lines on the Manhattan plots are the Bonferroni multiple test threshold corresponding type I error of 1%. The red lines on the QQ plots indicate the null hypothesis of no true association. Deviation from the expected P-value distribution is evident only in the tail area for each trait, suggesting that population stratification was adequately controlled. In total, these GWAS identified 27 SNPs significantly associated with HG and HH. The genes listed at the top of the Manhattan plots are reported as known genes which are highly related to growth or body size traits.

In our study, EBVs for HH and HG at 6, 12, 18, and 24 months old of the 3,325 cattle all passed the Shapiro-Wilk normality test and were used as eight traits in the GWAS. The GWAS results for the eight HG and HH traits were illustrated by Manhattan plots and Quantile-Quantile (Q-Q) plots in Fig 2. The Q-Q plots revealed that the population stratification was well controlled (λ close to 1). In Manhattan plots, a total of 27 SNPs achieved genome-wide significance (p < 2.38×10^−7^) associated with at least one of the eight traits, with the p-value ranging from 2.14×10^−7^(BTB-00553140 for HH12) to 5.67×10^−13^(ARS-BFGL-NGS-83478 for HG24), and the MAF ranging from 0.08 (ARS-BFGL-NGS-36823) to 0.47 (BTB-01040075).

The 27 significant SNPs (listed in Table 4) were located on 14 chromosomes, that is, BTA 1, 3, 6, 8, 10, 11, 12, 14, 15, 19, 22, 25, 29 and X chromosome. On BTA6, four SNPs were detected to be significant, three of which were associated with traits HG12, HG18, HG24, respectively, and the other one (ARS-BFGL-NGS-79814) was related to both HH6 and HH18. Meanwhile, 4 SNPs on X chromosome were found significantly associated with HG6, HG12, HH18 and HH24, respectively.

**Table 4:** Significant SNPs identified for Heart Girth (HG) and Hip Height (HH) in Chinese Holstein.

SNP*	Chr	Position (bp)	MAF	HG				HH			
				6	12	18	24	6	12	18	24
ARS-BFGL-NGS-76122	1	151590393	0.38							1.04E-09	8.06E-08
ARS-BFGL-NGS-36823	3	26971936	0.08		4.51E-08						
ARS-BFGL-NGS-111370	3	27115580	0.19					4.24E-11		5.02E-08	
ARS-BFGL-NGS-110127	3	87809919	0.38								3.58E-08
BTA-77968-no-rs	6	18898455	0.08					3.30E-08			
BTB-00262834	6	73008129	0.14							1.61E-07	
ARS-BFGL-NGS-82167	6	98952769	0.41				3.32E-09				
ARS-BFGL-NGS-79814	6	108635821	0.30		2.09E-07	5.26E-08					
BTA-92628-no-rs	8	4546756	0.27					1.34E-07			
ARS-BFGL-NGS-39319	8	31409233	0.38					1.61E-07			
ARS-BFGL-NGS-8362	10	7943252	0.20						6.60E-08		
ARS-BFGL-NGS-33225	11	12478437	0.36								1.07E-07
Hapmap48235-BTA-17587	12	23340411	0.49				7.99E-09				
ARS-BFGL-NGS-70821	14	3078843	0.24							1.46E-08	
BTB-00553140	14	13051936	0.12						2.14E-07		
BTB-01912619	15	20712027	0.44							9.85E-08	
BTA-08811-rs29022367	15	36997905	0.27						1.27E-07		
ARS-BFGL-NGS-88748	19	62087156	0.22					5.15E-08			
ARS-BFGL-NGS-83478	22	9271442	0.15	6.71E-09	1.48E-10	1.38E-10	5.67E-13				
Hapmap47137-BTA-95505	25	8060061	0.45			1.25E-09				7.52E-09	
ARS-BFGL-NGS-13985	25	35071379	0.23							1.70E-07	2.42E-08
ARS-BFGL-NGS-1643	25	37783875	0.40						8.11E-08		
ARS-BFGL-NGS-38901	29	400533	0.24								1.40E-07
BTB-01040075	X	5644107	0.47							5.67E-09	
ARS-BFGL-NGS-14625	X	13611851	0.21	1.30E-08	1.26E-07						
ARS-BFGL-NGS-118669	X	105267785	0.19								3.05E-08
Hapmap58680-rs29019274	X	112742618	0.36								6.73E-10

* All the SNPs listed were genome-wide significant in at least one trait (Bonferroni cutoff = 2.38E-7).

The numbers in the column headings below the trait names indicate the age of measurement, e.g., HG6 = heart girth at 6 months.

Of these 27 SNPs, 7 were associated with HG and 21 were associated with HH, with one SNP Hapmap47137-BTA-95505 on BTA25 associated with both HG and HH at the age of 18 months. According to the four growth stages, 7 SNPs, 8 SNPs, 10 SNPs and 10 SNPs were found to be associated with body size at 6 months, 12 months, 18 months and 24 months, respectively. For each trait, at least 2 SNPs showed a significant association, especially HH18, which had 8 significantly relevant SNPs. Three HG-significant SNPs (ARS-BFGL-NGS-79814, ARS-BFGL-NGS-83478, and ARS-BFGL-NGS-14625) and three HH-significant SNPS (ARS-BFGL-NGS-76122, ARS-BFGL-NGS-111370, and ARS-BFGL-NGS-13985) were associated with more than one age category, especially ARS-BFGL-NGS-83478, which was the most significant SNP for all four HG traits.

On BTA3, three significant SNPs were detected. Two of them (ARS-BFGL-NGS-36823 and ARS-BFGL-NGS-111370) were only 144 kb away from each other but were not in a strong LD (r^2^ = 0.01). The two SNPs were associated with HG (HH12) and HH (both HH6 and HH18), respectively. The other faraway SNP was associated with HH at 18 months old.

### Identification and annotation of candidate genes

Candidate regions were defined as a ±200-kb distance from the significant SNPs identified by GWAS in current study. Then, 62 genes locating within or overlapping the candidate regions of 23 SNPs were discovered. Nevertheless, none known gene within candidate regions of the rest 4 SNPs were found, for which the nearest genes along the chromosome were listed instead. In total, 66 candidate genes were determined and presented in Table 5. Among these candidates, 22 genes were associated with HG, mainly distributed in 6 chromosomes, that is, BTA3, 6, 12, 22, 25 and X chromosome. Besides, 46 candidate genes were related to HH, locating on 12 chromosomes, including BTA1, 3, 6, 8, 10, 11, 14, 15, 19, 25, 29 and X chromosome. Two genes, ATPase Na+/K+ transporting subunit alpha 1 (*ATP1A1*) on BTA3 and calcium-regulated heat stable protein 1 (*CARHSP1*, also called *CRHSP24*) on BTA25, were associated with both HG and HH.

**Table 5:** List of candidate genes associated with Heart Girth (HG) and Hip Height (HH) in Chinese Holstein.

Gene	Chr	Pos	Related SNPs	Associated traits
*DYRK1A*	1	151320007–151414207	ARS-BFGL-NGS-76122	HH18, HH24
*ATP1A1*	3	27002624–27035616	ARS-BFGL-NGS-36823, ARS-BFGL-NGS-111370	HG12, HH6, HH18
*IGSF3*	3	26691950–26817441	ARS-BFGL-NGS-36823	HG12
*MYSM1*	3	87924698–87966272	ARS-BFGL-NGS-110127	HH24
*JUN*	3	87841042–87843089	ARS-BFGL-NGS-110127	HH24
*PAPSS1*	6	18740229–18854670	BTA-77968-no-rs	HH6
*CEP135*	6	73021614–73095955	BTB-00262834	HH18
*EXOC1*	6	72512587–72989476	BTB-00262834	HH18
*HNRNPDL*	6	98983517–98990087	ARS-BFGL-NGS-82167	HG24
*HNRNPD*	6	98916663–98933786	ARS-BFGL-NGS-82167	HG24
*TMEM150C*	6	99050255–99068749	ARS-BFGL-NGS-82167	HG24
*ENOPH1*	6	98990766–99029323	ARS-BFGL-NGS-82167	HG24
*HAUS3*	6	108508717–108523817	ARS-BFGL-NGS-79814	HG12, HG18
*PIGG*	6	108729415–108758212	ARS-BFGL-NGS-79814	HG12, HG18
*FGFRL1*	6	108689770–109135290	ARS-BFGL-NGS-79814	HG12, HG18
*MXD4*	6	108490181–108501582	ARS-BFGL-NGS-79814	HG12, HG18
*PDE6B*	6	108818296–108849731	ARS-BFGL-NGS-79814	HG12, HG18
*GALNTL6*	8	3816704–5330615	BTA-92628-no-rs	HH6
*LURAP1L*	8	31581488–31636215	ARS-BFGL-NGS-39319	HH6
*MPDZ*	8	31133716–31280540	ARS-BFGL-NGS-39319	HH6
*CRHBP*	10	8038070–8052119	ARS-BFGL-NGS-8362	HH12
*F2R*	10	7879496–7897151	ARS-BFGL-NGS-8362	HH12
*MRPL23*	10	8139772–8140292	ARS-BFGL-NGS-8362	HH12
*F2RL1*	10	7956084–7965803	ARS-BFGL-NGS-8362	HH12
*S100Z*	10	7992986–7995677	ARS-BFGL-NGS-8362	HH12
*F2RL2*	10	7777598–7788321	ARS-BFGL-NGS-8362	HH12
*AGGF1*	10	8110496–8157658	ARS-BFGL-NGS-8362	HH12
*CYP26B1*	11	12371180–12387041	ARS-BFGL-NGS-33225	HH24
*STOML3*	12	23280345–23308336	Hapmap48235-BTA-17587	HG24
*NHLRC3*	12	23233220–23241391	Hapmap48235-BTA-17587	HG24
*TSNARE1*	14	3054763–3171547	ARS-BFGL-NGS-70821	HH18
MYC*	14	13769242–13774438	BTB-00553140	HH12
*ARHGAP20*	15	20907087–21120246	BTB-01912619	HH18
*RDX*	15	20589670–20657445	BTB-01912619	HH18
*FDX1*	15	20772619–20808021	BTB-01912619	HH18
*SOX6*	15	36600420–37082361	BTA-08811-rs29022367	HH12
*ABCA9*	19	62099290–62151581	ARS-BFGL-NGS-88748	HH6
*MGC134105*	19	62207474–62223857	ARS-BFGL-NGS-88748	HH6
ARPP21*	22	9674589–9681188	ARS-BFGL-NGS-83478	HG6, HG12, HG18, HG24
CARHSP1*	25	7734585–7746003	Hapmap47137-BTA-95505	HG18, HH18
*YWHAG*	25	34884279–34906639	ARS-BFGL-NGS-13985	HH18, HH24
*LRWD1*	25	35096009–35102381	ARS-BFGL-NGS-13985	HH18, HH24
*POLR2J*	25	35091302–35095680	ARS-BFGL-NGS-13985	HH18, HH24
*UPK3B*	25	35018574–35023605	ARS-BFGL-NGS-13985	HH18, HH24
*UPK3BL*	25	35034017–35039183	ARS-BFGL-NGS-13985	HH18, HH24
*DTX2*	25	34979329–35015686	ARS-BFGL-NGS-13985	HH18, HH24
*ORAI2*	25	35114124–35126594	ARS-BFGL-NGS-13985	HH18, HH24
*ZP3*	25	34960637–34968378	ARS-BFGL-NGS-13985	HH18, HH24
*PRKRIP1*	25	35147760–35173861	ARS-BFGL-NGS-13985	HH18, HH24
*SSC4D*	25	34930217–34940479	ARS-BFGL-NGS-13985	HH18, HH24
*ALKBH4*	25	35102455–35109899	ARS-BFGL-NGS-13985	HH18, HH24
*TRRAP*	25	37807383–37895047	ARS-BFGL-NGS-1643	HH12
*TMEM130*	25	37902689–37918785	ARS-BFGL-NGS-1643	HH12
*KPNA7*	25	37664542–37691692	ARS-BFGL-NGS-1643	HH12
*ARPC1A*	25	37564390–37587556	ARS-BFGL-NGS-1643	HH12
*PANX1*	29	590732–644582	ARS-BFGL-NGS-38901	HH24
ZBTB33*	X	5043333–5050947	BTB-01040075	HH18
*SASH3*	X	13681411–13695775	ARS-BFGL-NGS-14625	HG6, HG12
*XPNPEP2*	X	13645603–13672116	ARS-BFGL-NGS-14625	HG6, HG12
*UTP14A*	X	13790537–13807201	ARS-BFGL-NGS-14625	HG6, HG12
*OCRL*	X	13452405–13501005	ARS-BFGL-NGS-14625	HG6, HG12
*ZDHHC9*	X	13704736–13734121	ARS-BFGL-NGS-14625	HG6, HG12
*APLN*	X	13559123–13564931	ARS-BFGL-NGS-14625	HG6, HG12
*SMARCA1*	X	13358542–13433876	ARS-BFGL-NGS-14625	HG6, HG12
*MAOB*	X	105235855–105359155	ARS-BFGL-NGS-118669	HH24
*MAGEB16*	X	112749378–112760963	Hapmap58680-rs29019274	HH24

Chr: Chromosome

Pos: Position

* The nearest gene but not locating within the candidate region of the SNP.

Using these 66 putative genes, the potential pathway and gene-to-gene interaction network were constructed by IPA. Totally, 25 significant pathways were identified (p < 0.05) (S2 Fig), most of which were obvious pathways related to development and growth factors, such as G12/13 alpha signaling, p70S6K signaling, PDGF signaling.

**Fig 3:**
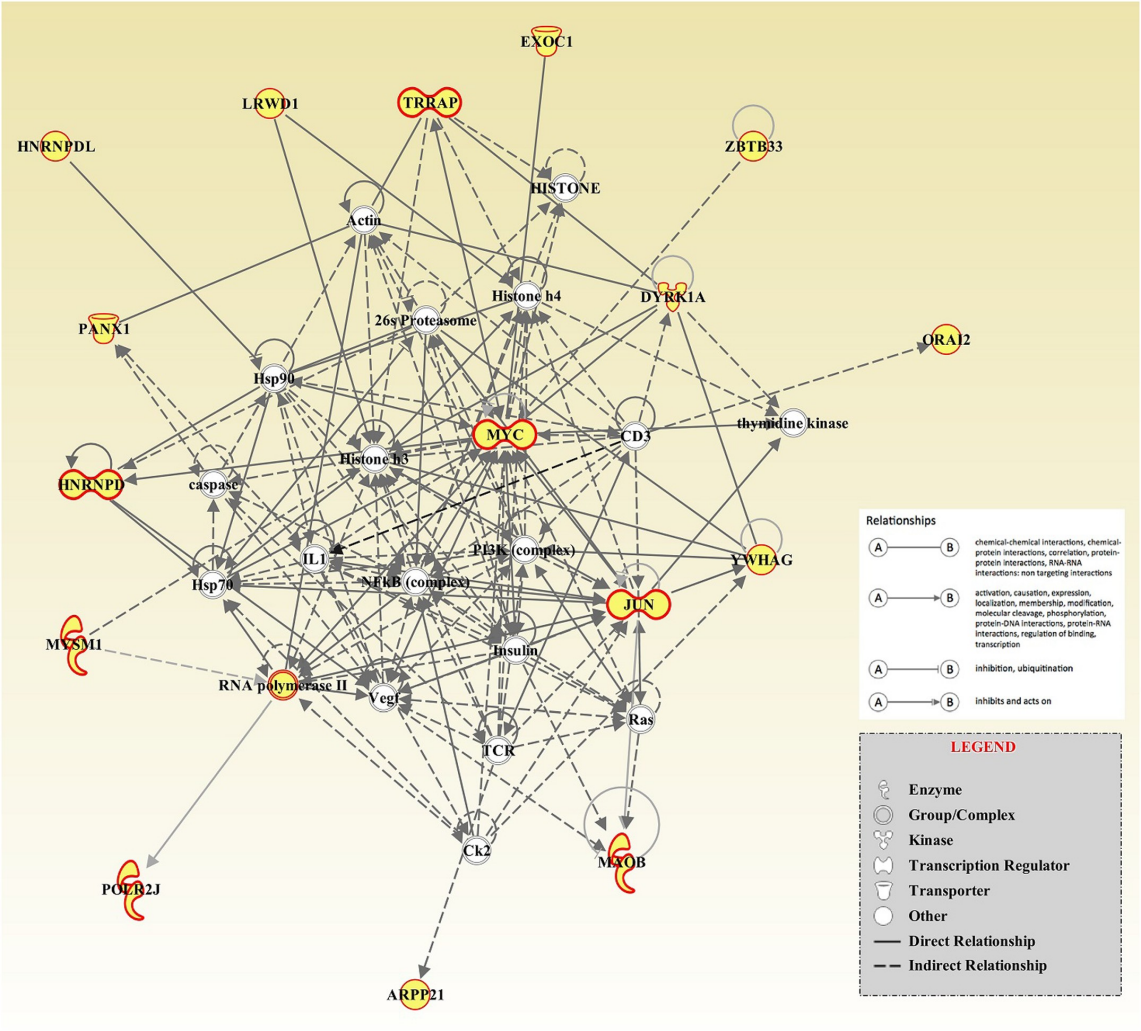
The related genes and their network with candidate genes. The network was constructed based on 16 candidate genes identified in current study by using Ingenuity Pathway Analysis (IPA) software. The IPA score was set at 34 for relating to cell cycle, connective tissue development and function, developmental disorder. The 16 candidate genes from current study were marked into grey, while the 18 white nodes are relevant transcription factors identified by IPA algorithm. Solid and dashed lines indicated direct interaction and indirect interaction, respectively. Genes are presented as nodes and their relationship are presented as edges. The functions of genes are illustrated by the shape of the node. The types of the relationship are illustrated by the ending shapes of the edges.

Seven relevant networks were constructed from these candidate genes for both HH and HG (S1 Table), and one interaction network of them containing 16 candidate genes had the high IPA score above 30 (IPA score = 34), which was related to developmental disorder as well as connective tissue development and function, with the v-myc avian myelocytomatosis viral oncogene homolog (*MYC*) gene at the center, including Jun proto-oncogene, AP-1 transcription factor subunit (*JUN*), dual specificity tyrosine phosphorylation regulated kinase 1A (*DYRK1A*), etc. (Fig 3).

Functional annotation results revealed more than 500 functions belonging to 255 categories from IPA (*p* < 0.05), out which 139 functions were annotated by two or more candidate genes. Developmental disorder and skeletal and muscular disorders were the two major function categories relating to body size, which contained 21 and 28 functions consisting of 16 and 18 genes, respectively (S2 and S3 Tables). Among these genes, 13 were both related to developmental disorder and skeletal and muscular disorders.

## Discussion

### Estimates of genetic parameters

Heritabilities of HG and HH at four growth stages obtained in this study were moderated to high estimates, which were in the range of previous estimates reported in the literature. For example, the heritability of HG was reported 0.22–0.38 in Holstein[4,28–31], and 0.35–0.47 in other breeds[29,32]; while the heritability of HH was 0.36–0.69 in Holstein[4,28,29,31,33], and 0.35–0.74 in other breeds[29,34].

It was well documented that the HG and HH had a medium correlation both genetically and phenotypically in bovine at adult stage[4,29,35,36]. Very few studies targeted on relationships between HG and HH at a younger age in cattle. A recent study reported a phenotypic (0.50) and a genetic (0.58) correlation between HG & HH at birth in Chinese Sanhe cattle[37], which fell into similar range with that in adulthood. In the Chinese Holstein, the phenotypic correlation between HG24 and HH24 was estimated to be 0.44, which was very close to an earlier report in adult Holstein in Netherlands(0.46)[4], and in adult Swiss Braunvieh (0.48) and Simmental (0.55)[35], as well as in adult Chinese Sanhe cattle (0.48). The genetic correlation between HG and HH in this study was 0.30–0.65, comparable with the reported range in dairy cattle (0.34–0.54)[4,29] and dual purpose cattle (0.47–0.58)[35–37].

### Candidate genes associated with HG and HH

In the present study, 66 genes nearby 27 significant SNPs were considered to be potential genes associated with body size traits. Among these genes, 22 locating on 6 chromosomes were associated with HG and 46 distributing on 12 chromosomes were associated with HH. Since these eight traits were not independent, it is expected that certain genes may be associated with two or more related body traits. 26 genes out of the 66 candidates were found regulating two or more traits, with two genes, *ATP1A1* and *CARHSP1*, being associated with not only HG but also HH. It is obvious that candidate genes discovered in this study relating to HH were even twice of those to HG.

All 66 candidate genes were used for pathway analysis and gene-to-gene interaction network construction, Totally 65 genes, except for *MGC134105*, which was failed to be annotated in IPA database, were enriched in functions related to developmental disorder and skeletal abnormalities and were constructed to seven relevant networks (S1 Table). Furthermore, we compared candidate genes detected in our study with previously revealed genes and genomic related information and found nine out of theses 66 candidate genes were already reported relevant to body size, growth and development in cattle, human, mice, and/or other animal species, which would be discussed following.

Three genes were related to HH12. The first one, transmembrane protein 130 (*TMEM130*) on BTA25 was also detected by Wu *et al*[38], that was associated with animal size and capacity in Chinese Holstein. Meanwhile, this gene is within a QTL region affecting calf size reported in Danish Holstein cattle[39]. In 2014, Zhao *et al*[40] has identified a selective signature in seven dairy and beef cattle breeds. For *TMEM130* is one of the poorly characterized genes[41], the function of this gene was still unknown. The second one was *MYC* on BTA14. *MYC* was suggested as a crucial mediator of signals that determine organ and body size in mammals[42]. And as it was also shown in Fig 3, *MYC* cooperated with most of the genes in the significant network. In human, it was also reported that a gene implicated in c-myc regulation, *FUBP3*, was associated with height[43]. The third gene *SOX6* (SRY-box 6) on BTA 15, was known to be important for cartilage formation[44] and skeletal development[45]. Deng *et al*. showed that *SOX6* is a direct target of *Yap1* for regulating chondrocyte differentiation in both skeletal development, postnatal growth, and bone repair[46]. And in human, SNPs in SOX6 were demonstrated to be significantly associated with the bone mass index and hip bone mineral density[47]. In chicken, a selective signature was detected maybe due to its essential roles in growth, development and reproduction[48,49].

Four genes were associated with HH24. The first one was *DYRK1A* on BTA 1, which was simultaneously related to HH18, was thought to be a causative factor in Down syndrome in human[50,51]. Point mutations in *DYRK1A* have been shown to be responsible for lower height. It was also documented in mice that mutations in *DYRK1A* could cause a decrease in body length and weight from birth to adulthood[52]. Furthermore, a selection signature was detected on this gene in European and African cattle populations, which explained the differences in the body size[53]. The second gene *JUN*, locating less than 32 kb from the significant SNP ARS-BFGL-NGS-110127 on BTA3 was reported to be required for axial skeletogenesis by regulating notochord survival and intervertebral disc formation in mice[54]. The third gene was Centrosomal protein 135 (*CEP135*) on BTA6, which is known as a centrosomal component and plays an important role in promoting centriole assembly and stability[55]. It was reported that *CEP135* was one of the MCPH (autosomal recessive primary microcephaly; MIM251200) genes, and pathogenic mutations in this gene caused autosomal recessive primary microcephaly and reduced the growth rate in human, characterized by reduced brain and skull size with sloping forehead and short stature[56,57]. The fourth *CYP26B1* (cytochrome P450, family 26, subfamily B, polypeptide 1) on BTA11, is one of the three CYP26 gene isoforms[58].*CYP26B1* was revealed to be responsible for the normal function of the growth plate and growing bones in zebrafish[59,60], mice[61] and human[62].

Furthermore, fibroblast growth factor receptor-like 1 (*FGFRL1*) on BTA6 was detected associated with HG12 and HG18. *FGFRL1* is the most recently discovered member of the FGFR family[63], which plays important roles in cell adhesion[64], embryonic development of slow muscle fibers[65], and bone formation[66]. In mice, this gene was preferentially expressed in skeletal tissues and skeletal malformations, including axial and appendicular skeletal anomalies[67], or a slight reduction in the overall size of the skeleton[68] were observed in the targeted *FGFRL1* knock-out mice. In human, Matoso *et al*. also reported that *FGFRL1* gene was associated with overgrowth[69]. Besides, *ATP1A1* on BTA3, as we aforementioned was associated with HG12, HH6 and HH18. *ATP1A1* encodes the α1 isoform, the major isoform of the α subunit of Na+/K+-ATPase[70]. Polymorphisms in *ATP1A1* could be potentially contributing to thermal tolerance[71], which further affected growth[72]. Additionally, Na+/K+-ATPase was proposed as a potential factor causing the unconventional secretion of Fibroblast Growth Factor 2 (FGF2)[70,73–75], which was known having profound effects on skeletal myoblasts proliferation in various animal systems. In chicken, selection for body size could significantly change the expression of *FGF2*[76,77]. And the abnormal of *FGF2* would cause a variety of skeletal malformations, including shortening and flattening of long bones and moderate macrocephaly[78] in mice.

## Conclusions

In total, 27 SNPs targeting to 66 candidate genes were identified to be associated with HG and HH at 6, 12, 18 and 24 months. Since many complex traits have a similar architecture across different species[21], we have made an attempt to compare some of our GWAS candidate genes with the previous reports about the same genes and their association with growth. Our findings confirmed nine known genes involving in development or associated with body size in cattle, human and mice studies. We also identified new genes through network and function analyses, including *ARPP21*, *AGGF*, *ZDHHC9*, *PDE6B*, *PIGG*, *MPDZ*, *OCRL*, *F2R*, *APLN*, *F2RL1*, *TRRAP*. Further investigations were required to identify or confirm the functions of these candidate genes, especially in relation to growth in dairy cattle.

## Supporting information

S1 Fig Scatter plots and fitting curves of growth traits. The growth traits are measured as hear girth (HG) displayed on the top panel and hip height (HH) on the bottom panel. The left panel illustrates the raw measurements over actual age that the measurements are taken. The dots in blue are accepted for the adjustment on age. The dots in yellow are removed for the adjustment. The distributions of adjusted measurements on age are displayed as blue dots on the right panel. The fitting curves (red curves) are derived from all the raw measurements, including both the blue and yellow dots.
(TIF)

S2 Fig Pathways identified in Ingenuity Pathway Analysis (IPA). The significances of the pathways are indicated by the bars as the negative log *p* values calculated by Fischer's test. In total, 25 pathways were identified at threshold of *p* < 0.05 indicated by the yellow straight horizontal line. Most of the pathways are related to development and growth factors, such as G12/13 alpha signaling, p70S6K signaling, and PDGF signaling. The bars are shaded into gray for pathways where predictions are not currently possible in IPA. The yellow line with squares presents the ratio of the candidate genes found by current study to the total number of genes within each pathway. The scale of the ratio is indicated by the secondary vertical axis on the right.
(TIF)

S1 Table Gene interaction networks constructed by IPA based on the candidate gene list. (DOCX)

S2 Table The 21 developmental disorder related diseases and bio functions identified by IPA. (DOCX)

S3 Table Skeletal and muscular disorders related 28 diseases and bio functions identified by IPA. (DOCX)
