# Supplementary figures and images for: Genome-wide association studies identified multiple genetic loci for body size at four growth stages in Chinese Holstein cattle
Source: PLoS One. 2017 Apr 20;12(4):e0175971. doi: 10.1371/journal.pone.0175971 (PMC5398616; doi:10.1371/journal.pone.0175971)

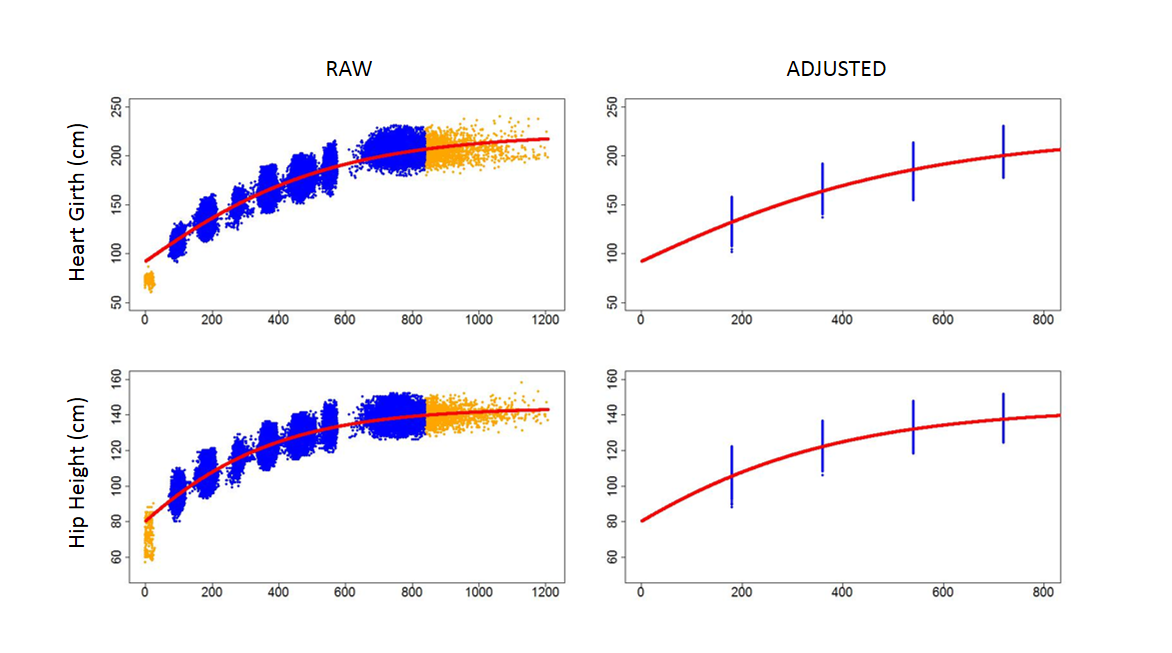

Supplement: S1 Fig — The growth traits are measured as hear girth (HG) displayed on the top panel and hip height (HH) on the bottom panel. The left panel illustrates the raw measurements over actual age that the measurements are taken. The dots in blue are accepted for the adjustment on age. The dots in yellow are removed for the adjustment. The distributions of adjusted measurements on age are displayed as blue dots on the right panel. The fitting curves (red curves) are derived from all the raw measurements, including both the blue and yellow dots. (TIF) [file pone.0175971.s001.tif]

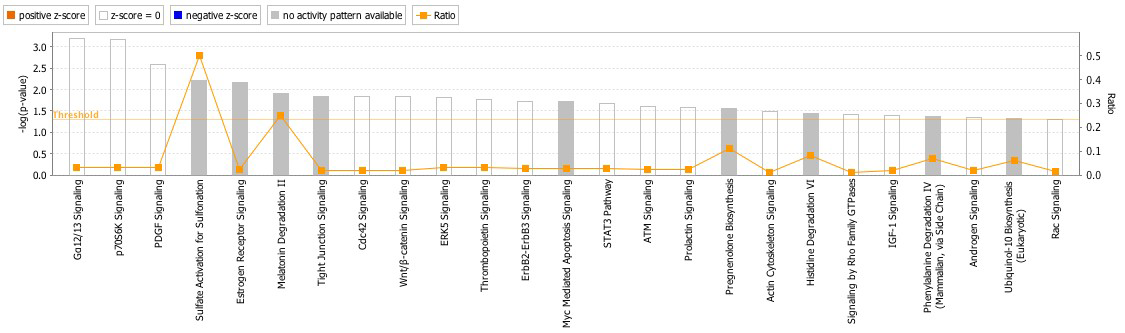

Supplement: S2 Fig — The significances of the pathways are indicated by the bars as the negative log p values calculated by Fischer's test. In total, 25 pathways were identified at threshold of p < 0.05 indicated by the yellow straight horizontal line. Most of the pathways are related to development and growth factors, such as G12/13 alpha signaling, p70S6K signaling, and PDGF signaling. The bars are shaded into gray for pathways where predictions are not currently possible in IPA. The yellow line with squares presents the ratio of the candidate genes found by current study to the total number of genes within each pathway. The scale of the ratio is indicated by the secondary vertical axis on the right. (TIF) [file pone.0175971.s002.tif]
