# Supplementary material for: Genome-wide association studies identified multiple genetic loci for body size at four growth stages in Chinese Holstein cattle
Source: PLoS One. 2017 Apr 20;12(4):e0175971. doi: 10.1371/journal.pone.0175971 (PMC5398616; doi:10.1371/journal.pone.0175971)
Supplement: S1 Table — (DOCX) [file pone.0175971.s003.docx]

**S1 Table. Gene interaction networks constructed by IPA based on the candidate gene list.**

| **Functions** | **Score** | **Candidate Gene^*^** |
| --- | --- | --- |
| Cell Cycle  Connective Tissue Development and Function  Developmental Disorder | 34 | *ARPP21,* ***DYRK1A****, EXOC1, HNRNPD, HNRNPDL,* ***JUN****, LRWD1, MAOB,* ***MYC****, MYSM1, ORAI2, PANX1, POLR2J, TRRAP, YWHAG, ZBTB33* |
| Organ Development  Cellular Development  Connective Tissue Development and Function | 28 | *APLN, ARPC1A,* ***ATP1A1****, F2R, F2RL1, F2RL2, FDX1,* ***FGFRL1****, PDE6B, RDX, SMARCA1,* ***SOX6****, XPNPEP2, ZP3* |
| Hereditary Disorder  Neurological Disease  Organismal Injury and Abnormalities | 26 | *ABCA9, ALKBH4, ARHGAP20, CARHSP1, CRHBP, DTX2, IGSF3, MRPL23, MXD4, NHLRC3, SSC4D, TSNARE1, ZDHHC9* |
| Hematological System Development and Function  Organismal Functions  Cell Cycle | 23 | *AGGF1,* ***CYP26B1****, ENOPH1, LURAP1L, OCRL, PAPSS1, PRKRIP1, S100Z, SASH3,* ***TMEM130****, UPK3BL, UTP14A* |
| Cell-To-Cell Signaling and Interaction  Nutritional Disease  Psychological Disorders | 14 | ***CEP135****, HAUS3, KPNA7, MPDZ, PIGG, STOML3, TMEM150C, UPK3B* |
| Organismal Injury and Abnormalities  Reproductive System Disease  Cardiovascular Disease | 2 | *GALNTL6* |
| Cancer  Organismal Injury and Abnormalities  Reproductive System Disease | 2 | *MAGEB16* |

^*^ *MGC134105* was failed to be annotated in IPA database.

The 9 candidate genes in bold were reported to be significantly associated with the growth and development in human, mice, and/or other animal species.
