# Supplementary material for: Genome-wide association studies identified multiple genetic loci for body size at four growth stages in Chinese Holstein cattle
Source: PLoS One. 2017 Apr 20;12(4):e0175971. doi: 10.1371/journal.pone.0175971 (PMC5398616; doi:10.1371/journal.pone.0175971)
Supplement: S2 Table — (DOCX) [file pone.0175971.s004.docx]

**S2 Table. The 21 developmental disorder related diseases and bio functions identified by IPA.**

| **Functions** | **Candidate Gene** |
| --- | --- |
| Abnormalities; Cancer; Cardiovascular Disease; Cardiovascular System Development and Function; Cellular Development; Connective Tissue Disorders; Developmental Disorder; DNA Replication; Embryonic Development; Gastrointestinal Disease; Hematological Disease Metabolic Disease; Neurological Disease; Ophthalmic Disease; Organismal Development; Organismal Injury and Hereditary Disorder; Recombination and Repair; Renal and Urological Disease; Reproductive System Disease; Skeletal and Muscular Disorders; Tissue Morphology | *AGGF1, APLN* ***CEP135****,* ***CYP26B1****,* ***DYRK1A****,* *F2R, F2RL1,* ***FGFRL1****,* ***JUN****, MPDZ,* ***MYC****, OCRL, PDE6B, PIGG, TRRAP, ZDHHC9* |

The 6 candidate genes in bold were reported to be significantly associated with the growth and development in human, mice, and/or other animal species.
