# Supplementary material for: Genome-wide association studies identified multiple genetic loci for body size at four growth stages in Chinese Holstein cattle
Source: PLoS One. 2017 Apr 20;12(4):e0175971. doi: 10.1371/journal.pone.0175971 (PMC5398616; doi:10.1371/journal.pone.0175971)
Supplement: S3 Table — (DOCX) [file pone.0175971.s005.docx]

**S3 Table. Skeletal and muscular disorders related 28 diseases and bio functions identified by IPA.**

| **Functions** | **Candidate Gene** |
| --- | --- |
| Cardiovascular Disease; Cardiovascular System Development and Function; Cell Cycle; Cell Morphology; Cellular Growth and Proliferation; Cellular Development; Connective Tissue Development and Function; Connective Tissue Disorders; Developmental Disorder; Embryonic Development; Gastrointestinal Disease; Hereditary Disorder; Inflammatory Disease; Inflammatory Response; Neurological Disease; Organ Morphology; Organismal Injury and Abnormalities; Organismal Development; Organ Development; Psychological Disorders; Skeletal and Muscular Disorders; Skeletal and Muscular System Development and Function; Tissue Development; Tissue Morphology | *AGGF1, APLN, ARPP21,****CEP135****,* ***CYP26B1****,* ***DYRK1A****, F2R, F2RL1,* ***FGFRL1****, HNRNPDL,* ***JUN****, MAOB, MPDZ,* ***MYC****, PANX1, PDE6B,* ***SOX6****, ZDHHC9* |

The 7 candidate genes in bold were reported to be significantly associated with the growth and development in human, mice, and/or other animal species.
